# Altering VP1 and VP2 expression in trans affects the transduction efficiency of AAV9

**DOI:** 10.3389/fbioe.2026.1753246

**Published:** 2026-02-26

**Authors:** Maxim K. Efremov, Alima Galieva, Andrew N. Brovin, Natalia V. Mesonzhnik, Mikhail B. Afonin, Elena N. Subcheva, Alexander Karabelsky

**Affiliations:** 1 Center for Translational Medicine, Gene Therapy Department, Sirius University of Science and Technology, Sirius, Russia; 2 Resource Centre of Analytical Methods, Laboratory Complex, Sirius University of Science and Technology, Sirius, Russia

**Keywords:** aav, adeno-associated virus, capsid engineering, capsid stoichiometry, gene therapy, transduction efficiency

## Abstract

Currently, adeno-associated virus (AAV) is one of the most reliable carrier for gene delivery in both proliferating and non-proliferating cells. Stable and long-lasting transgene expression has made this viral vector a key platform for the development of advanced therapy. Nevertheless, the widespread clinical use of AAV-based drugs remains limited due to their immunogenicity, low capsid capacity, and restricted tissue tropism. Tissue tropism depends largely on the the transduction efficiency of AAV capsids. In this study, we modified the standard three-plasmid transfection protocol to provide independent expression of VP1 or VP2 proteins from separate plasmids. Adjusting the ratio of these plasmids in the transfection mixture enabled alteration of the stoichiometric composition of the capsids, as SDS-PAGE and mass spectrometry confirmed. Increasing the amount of VP1 or VP2 in the capsid composition enhanced transduction efficiency, as demonstrated *in vitro* experiments on HEK293 cells. Obtained results contribute to a more comprehensive understanding of the AAV biology and have perspective of application in gene therapy.

## 1 Introduction

Gene therapy with adeno-associated viral (AAV) vectors is a rapidly developing field in modern translational medicine. Currently, more than 100 AAV-based drug prototypes are undergoing clinical trials, while seven have been already approved by the FDA and EMA for therapeutic use (FDA, 2025).

However, gene therapy employing AAV still has drawbacks, such as vector immunogenicity (Pipe et al., 2023), low capsid capacity (Chamberlain et al., 2016), and inadequate tissue specificity (Mulcrone et al., 2023). AAV serotype 9 (AAV9) has broad tissue tropism and the ability to transduce nerve cells (Zincarelli et al., 2008), enabling its application in the treatment of neurodegenerative (Saraiva et al., 2016; Dayton et al., 2012) and muscular (Vita et al., 2019; Jablonka et al., 2022) diseases. Nevertheless, the enhancement of its tissue specificity remains a pressing issue, as this would reduce the therapeutic dose and minimize side effects (Chand et al., 2021; Ertl, 2022; Pipe et al., 2023). Various strategies like swapping of promoter region, addition of regulatory elements and engineering of the capsid structure could modulate AAV infectivity (Wagner et al., 2021). Capsid modification is one of the most perspective methods for increasing transduction efficiency as the virus entry into the cell is the most challenging stage of gene delivery (Meyer and Chapman, 2022).

AAV capsid assembly is a stochastic process that occurs through the random incorporation of 60 viral proteins (VP) capsid proteins from a mixed pool, with an approximate ratio of 1:1:10 (VP1:VP2:VP3) (Wörner et al., 2021). Three VP proteins are encoded by the *cap* gene and are formed through alternative splicing with the utilization of alternative start codon. The VP3 sequence (59–61 kDa) is a common part of each VP protein. VP2 (64–67 kDa) is an elongated VP3, and its N-terminal region is known as the common VP1/VP2 region. VP1 (79–82 kDa) is approximately 137 amino acids longer than VP2, and this region is called the unique VP1 region (VP1u). VP3 forms the bulk icosahedral structure of AAV capsids. Furthermore, studies on AAV2 revealed that the formation of AAV-like particles is possible in the presence of VP3 alone (Warrington et al., 2004). However, VP1u contains a phospholipase A2 domain, and VP1u and VP1/VP2 contain a nuclear localization signal. These domains play a crucial role in the intracellular trafficking and endosomal escape, nuclear localization and genome release (Agb et al., 2012). In this regard, an increased proportion of VP1 and VP2 in the capsid composition may improve transduction efficiency of AAV.

In this study, we enabled the expression of VP1 and VP2 proteins *in trans*. By adjusting the ratio of plasmids in the transfection mixture, we altered the stoichiometry of the capsid. As a control, we produced standard AAV9 capsids using the traditional three-plasmid protocol with pAAV-GOI (gene of interest), pHelper, and pRepCap plasmids. Meanwhile, to make capsids with an altered VP ratio, we used a four-plasmid protocol with two pRepCap plasmids. We demonstrated that increasing the relative molar content of VP1- or VP2-encoding plasmids in the transfection mixture results in capsid formation with altered VP1:VP2:VP3 stoichiometry and enhanced transduction efficiency compared to wild-type capsids.

## 2 Materials and methods

### 2.1 Plasmid design

The M1L and T138A substitutions were introduced by site-directed mutagenesis of the entire pAAV2/9n plasmid (AddGene, United States) using a forward primer (5′-CTT​GCT​GCC​GAT​GGT​TAT​C-3′) and a reverse primer (5′-ACC​TGA​TTT​AAA​TCA​TTT​ATT​GTT​C-3′) and a forward primer (5′-GCT​GCT​CCT​GGA​AAG​AAG​AGG​CC-3′) and a reverse primer (5′-CTT​AGC​CGC​TTC​CTC​AAC​C-3′), respectively.

The M203L substitution was introduced in two stages. To do this, at first, two partially overlapping fragments were generated in the mutated region using forward (5′-AGT​CAG​TTG​CGC​AGC​CAT​CGA​C-3′) and reverse primers (5′-CAC​CAC​CTG​AAG​CAA​GTG​TAA​GAG​ATC​C-3′) or forward (5′-GAT​CTC​TTA​CAC​TTG​CTT​CAG​GTG​GTG​GC-3′) and reverse (5′-CGT​GAC​CTC​TAA​TAC​AGG​ACC​TCT​AGT​C-3′) primers. Obtained fragments were then joined with assembly PCR and resulting sequence was cloned into the pAAV2/9n plasmid, which already contained the M1L or T138A substitutions, via the AatII and SbfI restriction sites.

### 2.2 Cell lines

HEK293 suspension cells (ECACC 85120602) maintained in EmCD HEK293 Plus Medium (Eminence, PRC) were used for capsid production. Adherent HEK293T cells were maintained in DMEM High Glucose (4,500 mg/L), 2.5 mM L-glutamine, supplemented with 5% FBS. All cells were grown in a humidified incubator at 37 °C, 5% CO_2_ until approximately 80% confluence was reached.

### 2.3 Transfection

The following plasmids were used to generate AAV vectors: pHelper (Cell Biolabs, United States of America), pAAV2/9n (AddGene, United States of America), pAAV-CMV-GFP (Cell Biolabs, United States of America).

HEK293 suspension cells were seeded at a concentration of 5·10^5^ cells/mL in 900 mL of EmCD HEK293 Plus Medium. Transfection was performed after 24 h, when the cell concentration reached 1·10^6^ cells/mL. PEI MAX (Polysciences, United States) was used as the transfection agent at a DNA:PEI mass ratio of 1:5. The total DNA load was 1.5 μg per 10^6^ cells. To obtain wild-type AAV9, a molar ratio of pAAV-CMV-GFP:pHelper:pAAV2/9n of 2:2:5 was used. To produce capsids with an altered VP stoichiometric composition, the following molar ratios were used: pAAV-CMV-GFP:pHelper:pAAV2/9n (VP1orVP2):pAAV2/9n (VP2/3orVP1/3) at ratios of 2:2:2:2.5, 2:2:2:5 and 2:2:2:10, respectively. The cells were then incubated in a Multitron shaker incubator (INFORS, Switzerland) for 5 days at 37 °C, with 5% CO_2_, 80% humidity and 100 rpm. Details of plasmid design can be found in Figures 4A,B. Transfection scheme is depicted in Figure 4C.

### 2.4 AAV isolation and purification

The cells were lysed by adding Tween 20 (Sigma-Aldrich, United States of America) to a final concentration of 0.05%. The cells were then incubated in a shaker under the same conditions for 1 hour. Next, MgCl_2_ was added to the lysate to a final concentration of 1 mM, along with benzonase (Diaem, Russia) (30 U/mL). Incubation with shaking continued for another hour. The lysate was then centrifuged for 10 min at 3,000 g and clarified using a vacuum filtration system (TPP, Switzerland), with diatomite (Sigma-Aldrich, United States of America) added at a concentration of 1.5 g per 100 mL of lysate. The samples were then concentrated to a volume of 50 mL using a Vivaflow 200 HY ultrafiltration system (Sartorius, Germany) and a Masterflex 77,921–65 L/S peristaltic pump (Masterflex, Germany).

The AAV capsids were chromatographically purified using an Omnifit Adjustable/1 chromatography column (Diba Kinesis, United States of America) and a POROS CaptureSelect AAVX affinity sorbent (Thermo Fisher Scientific, United States of America), on a Quest 10 Plus chromatography system (Bio-Rad, United States of America). The following buffer solutions were used for purification: equilibration buffer (50 mM Tris-HCl, 0.05% Tween 20, 0.15 M NaCl, pH 8.0), washing buffer 1 (50 mM Tris-HCl, 1.5 M Urea, 5 mM EDTA, pH 8.0), washing buffer 2 (20 mM Tris-HCl, 0.05% Tween 20, pH 8.0), elution buffer (0.1 M Glycine-HCl, 0.05% Tween 20, 0.5 M L-Arginine, pH 2.0), regeneration buffer (8 M Urea, pH 1.5).

**FIGURE 1:**
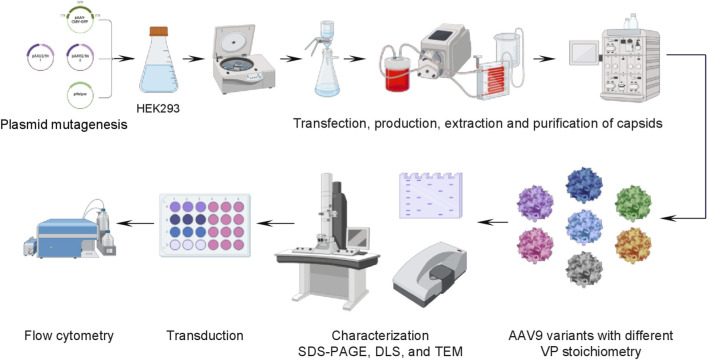
AAV9 production, purification and characterization workflow.

The eluates were then concentrated using JetSpin® centrifugal filters (Jet Biofil, China). The samples were washed twice with phosphate-buffered saline. After concentration, the capsids were resuspended in 1 mL of phosphate-buffered saline and passed through a 0.22 μm pore size syringe PES filter (Jet Biofil, China). The capsids preparations were stored in a freezer at −80 °C. Figure 1 shows the process flow diagram for the production and purification of AAV9 viral vectors.

### 2.5 Quantification of AAV viral genomes by qPCR

The quantitative determination of viral genomes concentration was performed using a real-time polymerase chain reaction on viral inverted terminal repeat sequences. Each sample was pre-treated with DNase I (Sintol, Russia) and proteinase K (Thermo Fisher Scientific, United States of America). Reaction mix was prepared with an HS-qPCR kit (Biolabmix, Russia) with a forward primer (5′-GGA​ACC​CCT​AGT​GAT​GGA​GTT-3′), reverse primer (5′-GGA​ACC​CCT​AGT​GAT​GGA​GTT-3′) and probe (5′-FAM-CACTCCCTCTCTGCGCGCTCG-BHQ2-3′) as manufacturer recommended. Serial dilutions of linearized pAAV-CMV-MCS (Cell Biolabs, United States of America) plasmid were used to set up a calibration curve. Amplification was performed using an Applied Biosystems StepOnePlus real-time detection amplifier (Thermo Fisher Scientific, United States of America). The results were analyzed using StepOnePlus software (Thermo Fisher Scientific, United States of America).

### 2.6 SDS-PAGE

The protein composition of the capsids was validated using SDS-PAGE (polyacrylamide gel electrophoresis under denaturing conditions) according to modified Laemmli method (LAEMMLI, 1970). Prior to application, 50 μL of each sample was incubated with Laemmli sample buffer (Bio-Rad, United States of America) for 5 min at 95 °C. Unstained protein standards (Bio-Rad, United States of America) were used as a marker. Electrophoretic separation was performed in a Tris-glycine buffer solution for 15 min at 100 V in a 4% stacking gel followed by 60 min at 150 V in 10% resolving gel. The gel was then washed three times in distilled water and stained with Coomassie G-250 dye (Bio-Rad, United States of America). Visualization and documentation of stained gel was made using the ChemiDoc Imaging System (Bio-Rad, United States of America). Densitometric evaluation was performed in ImageLab 6.1 software (Bio-Rad, United States of America).

### 2.7 DLS

The formation of capsid aggregates was evaluated using the dynamic light scattering method (DLS) with a Zetasizer Ultra particle size analyzer (Malvern Panalytical, United States) in a small-volume cuvette according to a previously described method (Tumaev et al., 2024). A 50 µL of each sample was taken for measurement. The data processing was performed using ZS XPLORER software (Malvern Panalytical, United States).

### 2.8 Transmission electron microscopy

Scanning transmission electron microscopy was used to evaluate the size, shape and ratio of empty and full capsids. It was performed using a Crossbeam 550 microscope (Carl Zeiss, Germany), equipped with a transmission electron microscopy detector, at an accelerating voltage of 30 kV. To ensure a uniform distribution of the capsids on the support grid surface, the grid was treated with an air plasma for 10 s using a Zepto plasma cleaning system (Diener Electronic, Germany). An aliquot of 10 μL of the capsid-containing sample was applied to the grid (230 mesh, carbon/formvar coating, EMCN, China), incubated for 2 min, washed with distilled water twice, and then contrasted with a 1% aqueous solution of uranyl acetate (Polysciences Inc., United States) and dried on air.

The relative proportion of full and empty viral particles was determined visually from STEM-micrographs using the following Equation 1:

$$\%F=\sum \text{Fn}/\left(\sum \text{Fn}+\sum \text{En}\right)\times 100$$ (1)

where %F represents the relative content of full capsids in the sample (expressed as a percentage), Fn is the absolute number of full viral capsids identified in n micrographs, and En is the absolute number of empty viral capsids in the same n micrographs. Broken capsids were excluded from the analysis, while partially filled capsids were counted as empty. The total number of viral particles was 219 for the AAV9-WT, 1,024 for the AAV-VP1-2 and 385 for the AAV9-VP2-2.

### 2.9 Transduction and flow cytometry

The adherent HEK293T cells were washed twice with PBS (VWR, United States of America), detached with 0.25% trypsin solution (PanEco, Russia), counted using automatic cell counter Countess II (Thermo Fisher Scientific, United States of America), diluted in DMEM High Glucose (4,500 mg/L), 2.5 mM L-glutamine, supplemented with 5% FBS and seeded to the wells of a 24-well plate at a concentration of 10^5^ cells per well. Capsids were then added at a dosage of 100,000 viral genome per cell (VG/cell). Assessment of the relative number of transduced cells was conducted 72 h after transduction using a CytoFLEX B2-R2-V0 flow cytometer (Beckman Coulter, United States of America). The cells were washed twice with PBS (VWR, United States of America), detached with 0.25% trypsin solution (PanEco, Russia) and resuspended in FACS buffer containing 2% FBS and 1 mM EDTA in PBS. Data were recorded using CytExpert 1.2 software for processing single live cells positive for FITC. Single FITC-positive cells were recorded. Analysis of the mean fluorescence intensity values in FITC-positive cell populations was performed using FlowJo™ v10 software.

### 2.10 Characterization of VP proteins by LC-MS/MS

Capsid samples were thawed to ambient temperature prior to analysis. To denature the capsids, formic acid (purity ≥99%, Sigma-Aldrich, United States of America) was added to the AAV samples at a concentration of 0.5%. The samples were then injected into the instrumental system at concentrations ranging from 1.9 · 10^11^ to 1.8 · 10^13^ VG/mL. The VP capsid proteins were analyzed using an Ultimate 3,000 Dionex system (Thermo Fisher Scientific, United States of America) for ultra-performance liquid chromatography (UPLC), coupled with a maXis 4G high-resolution mass spectrometer (Bruker, Billerica, United States of America) (resolution 60,000).

Protein separation was performed using an ACQUITY UPLC Peptide BEH C4 300 Å analytical column, 2.1 mm × 100 mm, particle size 1.7 μm (Waters, United States of America). A mixture of 0.1% formic acid and 0.05% trifluoroacetic acid (purity ≥99%, VWR, United States of America) in deionized water (18.2 MΩ cm, Merck Millipore, United States of America) was used as the mobile phase (MP) “A”. Mobile phase B (MP B): 0.1% aqueous solution of formic acid in acetonitrile (Fisher Chemical, United States of America). AAV VP(1–3) proteins were separated by gradient elution at a constant temperature of 60 °C: 0.0–2.0 min (5% MP B, 0.5 mL/min); 2.0–3.0 min (5%–25% MP B, 0.4 mL/min); 3.0–10.0 min (25%–50% MP B, 0.4 mL/min); 10.0–11.0 min (50%–95% MP B, 0.4 mL/min); 11.0–12.0 min (95%–95% MP B, 0.5 mL/min); 12.0–13.0 min (95%–5% MP B), 13.0 min–15.0 min (5%–5% PF “B”, 0.5 mL/min). The analytes were ionised using an electrospray ionisation (ESI) source in positively charged ions detection mode. The ion source parameters were as follows: a capillary voltage of 4.5 kV; a nebulizer pressure of 2.5 bar; a dry gas flow rate of 10 L/min (nitrogen); and a dry temperature of 350 °C. The end plate offset was set to 550 eV. The detector settings were optimised to ensure high sensitivity and resolution when analyzing high-molecular-weight compounds. The Funnel 1 RF and the multipole RF were set to 400 VPP and 800 VPP, respectively; the isCID energy was set to 5 eV; and the quadrupole ion energy was set to 7 eV. The collision energy was set to 5 eV, the collision RF to 1500 VPP, the ion transfer time to 70 μs and the pre-pulse storage to 45 μs.

Full scan data acquisition was performed by scanning from a mass range of 800–4,000 m*/z*. Spectra were recorded at a frequency of 1 Hz with active ion beam focusing option and averaged with a factor of 2. Both the linear and the profile spectra were stored, and the mass spectral peak detection was performed using the maximum intensity method. The absolute threshold was set to 25 cts and the peak summation width to 5 pts. The Q-TOF mass scale was externally calibrated using a commercially available calibration mixture from Agilent Technologies (ESI-L Low Concentration Tuning Mix, Agilent, United States of America). Data processing was performed using the Protein Metrics software (United States of America).

Mass spectra were deconvoluted using the Protein Metrics’ Intact Mass workflow. The average masses of VP1, VP2 and VP3 were calculated based on the amino acid sequence of the wild-type AAV9 proteins (UniProt: Q6JC40, Q6JC40_9VIRU) (UniProt, 2025). The capsid protein sequences were imported from the database in FASTA format, and the VP1 and VP3 sequences were considered for removal of the N-terminal methionine residue. Acetylation of the N-terminal region was set as a variable modification. Deconvolution parameters: the mass range was set from 20,000 to 100,000 Da, the *m/z* range from 800 to 4,000, the distance between charge vectors was 0.6, the baseline radius was 8.0 m*/z*, smoothing sigma was 0.001 m*/z*, step was 0.004 m*/z*, mass smoothing sigma was 7.0, mass step was 1.0, maximum number of iterations was 20, and charge range was from 5+ to 60+. The mass error tolerance after deconvolution was 20 ppm.

## 3 Results

### 3.1 AAV9 capsid assembly in the absence of or with independent expression of VP1 and VP2 proteins

According to the previously proposed model (Wörner et al., 2021), the relative expression levels of VP proteins predominantly determine the stoichiometry of the AAV capsid. Therefore, in order to produce capsids with different stoichiometric compositions, we obtained genetic constructs for independent expression of VP1 and VP2. Regulation of VP1 or VP2 content was achieved by co-transfection of varying ratio of plasmids pAAV2/9n-VP2/3 and pAAV2/9n-VP1 or pAAV2/9n-VP1/3 and pAAV2/9n-VP2.

**FIGURE 2:**
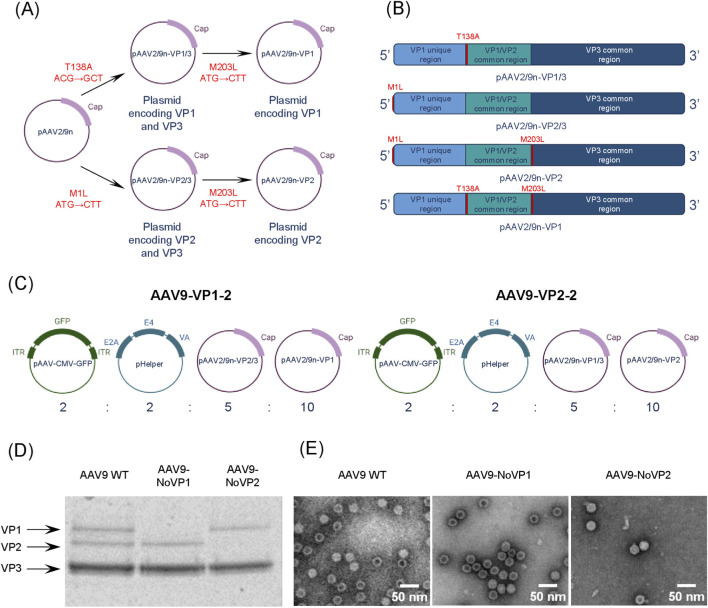
Independent expression of VP capsid proteins. **(A)** Mutagenesis scheme. **(B)** Schematic representation of the nucleotide sequences of the *cap* gene in the pAAV2/9n-VP1/3, pAAV2/9n-VP2/3, pAAV2/9n-VP2, and pAAV2/9n-VP1 constructs. Mutations are marked in red. **(C)** Scheme showing the plasmid ratios used in the transfection mixture to obtain capsids with altered stoichiometry. **(D)** Electrophoregram of AAV capsid proteins (10% SDS-PAGE). **(E)** Electron microphotographs of capsid preparations. Legend: AAV9 WT—wild-type capsids; AAV9-NoVP1 — capsids with VP1 knocked out; AAV9-NoVP2 — capsids with VP2 knocked out.

As VP protein expression involves the use of alternative start codons, mutations in these codons can specifically suppress the synthesis of a particular VP protein. Knockout of VP1, VP2 and VP3 expression was achieved by the M1L (ATG→CTT), T138A (ACG→GCT) and M203L (ATG→CTT) mutations, respectively (Figure 2B). In the first stage, we introduced the T138A and M1L substitutions to obtain the genetic constructs pAAV2/9n-VP1/3 and pAAV2/9n-VP2/3, which encode the proteins VP1/VP3 and VP2/VP3, respectively. Then, we introduced the M203L mutation into each of the sequences to obtain the constructs pAAV2/9n-VP2 or pAAV2/9n-VP1, encoding the VP2 or VP1 proteins exclusively (Figure 2A).

Previously, it has been shown that AAV2 capsids could be formed without VP1 and VP2 proteins (Warrington et al., 2004). We produced AAV9 viral particles by triple plasmid transfection, using pAAV2/9n-VP2/3 and pAAV2/9n-VP1/3 constructs as packaging plasmids to obtain capsids without VP1 and VP2, respectively. The capsids of both variants had a shape and size similar to wild-type AAV9 (Figure 2E), while SDS-PAGE analysis validated the absence of the respective proteins (Figure 2D). Thus, it was confirmed that the introduction of mutations into the start codons of the *cap* gene allows for the selective knockout of the synthesis of individual AAV9 capsid proteins.

Subsequently, AAV assembly was performed by the incorporation of a fourth plasmid encoding the lacking viral protein into the transfection mixture. It was demonstrated that the synthesis of all structural capsid proteins was provided by a four-plasmid transfection, whereby the pAAV2/9n-VP2/3 constructs were combined with the pAAV2/9n-VP1 and pAAV2/9n-VP1/3 with pAAV2/9n-VP2. The relative quantity of each protein was controlled by modifying the molar ratio of the plasmids. Consequently, the pAAV2/9n-VP2/3 (pAAV2/9n-VP1/3) and pAAV2/9n-VP1 (pAAV2/9n-VP2) constructs were co-transfected at a 1:2 ratio, thereby yielding AAV9-VP1-2 and AAV9-VP2-2 viral particles (Figure 2C).

### 3.2 Evaluation of the transducing capacity of AAV9 capsids expressing VP1 and VP2 *in trans*

**FIGURE 3:**
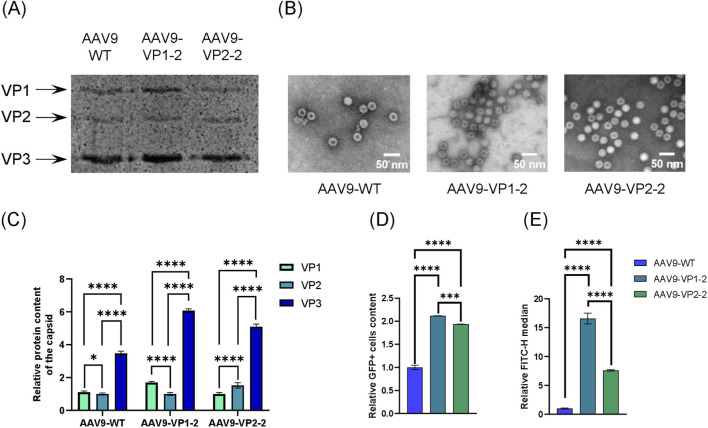
Characterization of capsids with altered stoichiometric composition. **(A)** Electrophoregram of AAV capsid proteins (10% SDS-PAGE). **(B)** Electron microphotographs of capsids with altered stoichiometric composition. **(C)** Relative content of VP1, VP2, and VP3 capsid proteins according to semi-quantitative densitometry (****) p-val <0.0001, (*) p-val = 0.0217 (two-way ANOVA, N = 15). **(D)** Relative content of GFP + HEK293 cells 72 h after transduction according to flow cytometry (dose of 100,000 viral genomes per cell) (****) p-val <0.0001, (***) p-val <0.001 (two-way ANOVA, N = 3). **(E)** Median fluorescence intensity values. (****) p-val <0.0001 (two-way ANOVA, N = 3). Legend: AAV9 WT—wild-type capsids; AAV9-VP1-2 — capsids with increased VP1 content; AAV9-VP2-2 — capsids with increased VP2 content.

As the HEK293 cell line is effectively transduced by AAV9 (Gurtsieva et al., 2024), it was selected for transduction at a dosage of 100,000 viral genomes per cell (VG/cell). Flow cytometry analysis was used to estimate relative content of live single GFP + cells in the total population of transduced cells (Supplementary Figure S1). We found that efficiency of transduction has a positive correlation with altering the amount of VP1 and VP2 plasmids in the transfection mixture. Compared to the wild-type capsid, AAV9-VP1-2 and AAV9-VP2-2 demonstrated 2.1 and 1.9 times increased percentage of transduced cells, respectively, with a high level of significance (p-value <0.005) (Figure 3D). The MFI values showed the same trend, with differences of 16.5- and 7.6-fold between the AAV9-VP1-2 or AAV9-VP2-2 and AAV9-WT (p-value <0.001) (Figure 3E).

### 3.3 Assessment of the protein composition of capsids using SDS-PAGE densitometry

In order to evaluate the effect of the plasmid ratio in the transfection mixture on the stoichiometric composition of the capsids, SDS-PAGE followed by semi-quantitative densitometry was used (Figure 3A). The comparison was made with wild-type AAV9 capsids. The statistical analysis was conducted using GraphPad Prism 8.2.1 employing a two-way ANOVA test to analyze the data. The VP1:VP2:VP3 ratio was approximately 1.6:1:6 for AAV9-VP1-2 and 1:1.5:5 for AAV9-VP2-2. A statistically significant increase in the relative content of VP1 and VP2 proteins was identified in the AAV9-VP1-2 and AAV9-VP2-2 capsid samples, respectively. In contrast, the VP1:VP2 ratio in wild-type capsids remained approximately equal to the classic 1:1 ratio.

### 3.4 Mass spectrometric analysis of capsid composition

**FIGURE 4:**
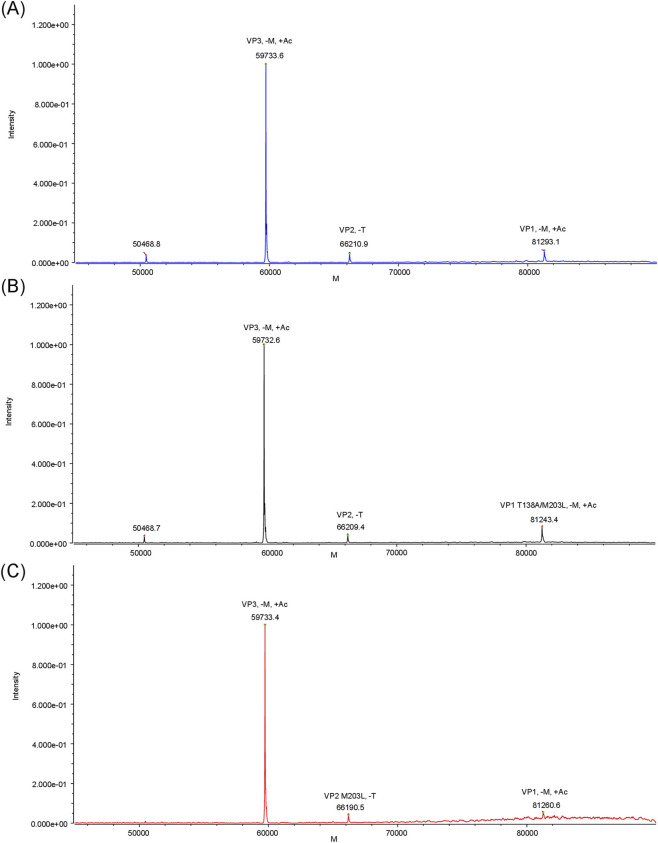
Deconvoluted mass spectra of VP proteins from wild-type AAV9 capsids **(A)**, AAV9-VP1-2 **(B)**, and AAV9-VP2-2 **(C)**.

MS-based analysis of wild-type AAV9 capsids revealed three major protein variants (Figure 4A). The mean molecular weights are presented in Table 1. The amino acid sequences of all VP structural proteins for the three capsid variants are shown in Supplementary Figure S2. The measured mass of 66,210.9 Da corresponds to the N-terminal sequence 139–736 VP2 (relative to the full-length VP1 protein), starting with alanine A139. The cleavage of the N-terminal threonine residue T138 for VP2 is consistent with the literature data (Zhou and Wang, 2023; Jin et al., 2017).

**TABLE 1:** The molecular masses of capsid proteins determined using LC-MS/MS.

Sample	Protein	Average molecular weight, da (error, da)	
		Calculated	Experimental
WT	VP1	81,290.5	81,293.1 (2.6)
	VP2	66,209.8	66,210.9 (1.2)
	VP3	59,732.8	59,733.6 (0.8)
AAV9-VP1-2	VP1_T138A_M203L	81,242.4	81,243.4 (1.0)
	VP2	66,209.8	66,209.4 (−0.4)
	VP3	59,732.8	59,732.6 (−0.2)
AAV9-VP2-2	VP1_ T138A	81,260.1	81,260.6 (0.5)
	VP2_M203L	66,191.8	66,190.5 (−1.2)
	VP3	59,732.8	59,733.4 (0.6)

The other two peaks in the deconvolution spectrum (Figure 4A), with average molecular weights of 81,293.1 Da and 59,733.6 Da (Table 1), correspond to the amino acid sequences 2–736 VP1 and 204–736 VP3, respectively, taking into account the removal of the N-terminal methionine residue (M1 and M203). Concurrently, the VP1 and VP3 peaks exhibited a shift from the calculated mass by approximately 42 Da, indicating a single acetylation of each protein. It should be noted that the removal of the N-terminal methionine residue of cellular proteins, including VP1 and VP3 of AAV capsids, is accompanied by acetylation of the subsequent alanine residue (A2 and A204) (Zhou and Wang, 2023; Jin et al., 2017; Hwang et al., 1979). Additional peak with a molecular weight of 50,468.8 Da (Figure 4A) in the deconvolution spectrum was identified as a truncated variant of the VP3 sequence, presumably formed as a result of hydrolytic cleavage of the polypeptide chain at the aspartic acid residue at position D658.

Deconvolution mass spectra of VP1 proteins from recombinant capsids, obtained by expressing VP1 and VP2 *in trans* with varying molar ratios of plasmids pAAV2/9n-VP2/3 (pAAV2/9n-VP1/3) and pAAV2/9n-VP1 (pAAV2/9n-VP2), revealed the presence of point amino acid substitutions in the initiation codons. As can be seen from the mass spectrometry data (Figure 4B), the deconvolution spectrum of the capsid proteins of the AAV9-VP1-2 sample shows three distinctive peaks, corresponding to VP1, VP2, and VP3. The experimentally measured molecular masses of VP2 and VP3 fully correspond to the calculated values for wild-type AAV9, while the mass of VP1 was reduced by 48 Da. This difference may be explained by the cumulative effect of two point mutations in the amino acid sequence: T138A (101.1 Da–71.1 Da = 30 Da) and M203L (131.2 Da–113.2 Da = 18 Da).

In case of AAV9-VP2-2, the deconvoluted mass spectrum is represented by a main VP3 peak, whose molecular weight corresponds to the calculated value for wild-type VP3 AAV9 (Figure 4C). The VP2 variant has a molecular weight reduced by ∼18 Da compared to wild-type AAV9 VP2, and the VP1 variant is ∼30 Da lower than the calculated value for wild-type AAV9 VP1. These mass difference correspond to the expected values for the VP2 M203L (131.2 Da → 113.2 Da) and VP3 T138A (101.1 Da–71.1 Da = 30 Da) mutations, confirming the specificity of the missense modifications made.

## 4 Discussion

In previous studies, it has been determined that the N-terminal regions of VP1 and VP2 contain a phospholipase domain and a nuclear localization signal (Agb et al., 2012). These extensions play crucial roles in endosomal trafficking and escape, nuclear localization, and viral genome release. This suggests that an increase in the relative content of VP1 and VP2 in the capsid composition may improve its transducing efficiency. This statement was recently confirmed in a study in which the relative content of VP1 and VP2 was increased by reducing the expression of VP3 (Onishi et al., 2023). The same study revealed a direct correlation between the ratio (VP1+VP2)/VP3 and transduction efficiency. Previously, superabundant VP1 rAAV capsids were formed with a VP1:VP2:VP3 protein ratio of 1.9:0.1:8 (Wang et al., 2017) using the VV-Ad system, which included two viral vectors. Vaccinia vector (VW22-PM) provided all Rep and Cap functions, while Ad/AAV hybrid vector provided the rAAV genome. Both vectors were utilized to transduce specially created QW158-7 cells with the E1a/E1b gene integrated into the genome. rAAVs produced with this system had an increased VP1 content and demonstrated effective *in vivo* transduction of retinal cells after intravitreal injection. However, this approach complicates both upstream and downstream processes, as it requires a special cell line, the construction of helper vectors, and further purification of samples from residual viral vectors. Therefore, we attempted to develop a method for increasing the VP1 content using traditional transient transfection, a technique that has proven to be both effective and safe.

In this study, we provided synthesis of each of the capsid proteins *in trans* using four-plasmid transfection. This approach differs from traditional three-plasmid transfection in that the plasmid encoding all the capsid proteins has been replaced by two separate plasmids each encoding one of these proteins. The produced capsids demonstrated a transduction efficiency twice that of the wild-type AAV9 control on HEK293 cells. Electron microscopy (Figure 3B) revealed that the capsids had an approximate diameter of 25 nm and exhibited a morphology analogous to that of the wild-type capsids. Although altering the stoichiometry of AAV VPs often leads to assembly impairment (Khanal et al., 2023), we did not detect partially assembled viral particles in the samples. However, according to the TEM data (Table 2), a decrease in the packaging efficiency of the capsids was revealed. This may be related to recent data (Dogbey and Barth, 2025) showing that an increase in VP1/VP2 content, accompanied by a decrease in VP3 content, results in a lower percentage of filled capsids. An increase in the VP1 or VP2 content of the capsids has also led to a slight increase in their tendency to form aggregates, as confirmed by DLS (Table 2).

**TABLE 2:** Relative content of aggregates, genomic titers and packaging efficiency of capsid samples.

Sample	Fraction size (DLS), nm	Relative fraction content (DLS), %	Genomic titers (qPCR), VG/mL	Packaging efficiency (TEM), %
AAV9-WT	23.97	98.3	4.4 · 10^12^	55
	407.3	1.7		
AAV9-VP1-2	24.08	97.49	2.83 · 10^11^	39
	286.4	1.82		
	4,979	0.69		
AAV9-VP2-2	23.86	98.38	1.9 · 10^11^	44
	387.9	0.89		
	5,048	0.73		

Notably, the start codon mutations M1, T138, and M203 used in our study had previously been tested in rational capsid engineering. For instance, to enhance transduction of FAP receptor-positive cells by the AAV-DJ capsid, independent expression of VP1 or VP2 conjugated with the FAP nanobody was employed (Hoffmann et al., 2024). This approach limited the inclusion of excess nanobodies in the capsid, avoiding potential steric hindrances to capsid formation. In another study, VP1 containing inserts of various protein domains was also expressed in trans (Hoffmann et al., 2023). Another interesting approach was utilised in the creation of protein carrier AAV (Hoffmann et al., 2025). In this study, independently expressed VP1 of the AAV-DJ capsid contained a green fluorescent protein nanobody (GFPnb) that specifically binds to GFP and ensures its packaging into the capsid. In the study (Lux et al., 2005), VP2 was fused to the N-terminus of GFP to visualize viral trafficking. It should also be noted that modifications to the N-terminus of VP2 often alter the amino acid sequence of VP1 (Warrington et al., 2004; Dogbey et al., 2025), whereas the use of four-plasmid transfection can avoid this.

It is important to note that two non-structural proteins necessary for AAV assembly, AAP and MAAP, are encoded in the alternative reading frame inside the *cap* gene (Kuz et al., 2024). The introduced substitutions resulted in simultaneous alterations in the amino acid sequences of these proteins. Thus, the mutation (ACG→GCT), leading to the T138A substitution in the VP1 and VP2 proteins, simultaneously causes R112L substitution in MAAP. Likewise, the mutation (ATG→CTT), resulting in the M203L substitution in VP1, VP2 and VP3, also leads to the W28L substitution in AAP. Therefore, both the native forms of MAAP and AAP and their mutant variants are involved in capsid assembly when producing the AAV9-VP1-2 and AAV9-VP2-2 variants. Since the role of these proteins in capsid assembly is not yet fully understood (Kuz et al., 2024), it can be hypothesized that the mutations that have arisen in them may affect their function.

For example, it is known that the R112 position is part of the basic amino acid-rich cluster of the MAAP protein. As this region is believed to act as a nuclear localisation signal (Aksu et al., 2024), changes to its structure could result in impaired nuclear localisation of MAAP. It is also known that deleting the C-terminal fragment of MAAP can slow down capsid degradation (Galibert et al., 2021). Additionally, the positive charge of the C-terminus of MAAP is thought to enable binding to negatively charged DNA, thereby regulating DNA packaging into capsids (Galibert et al., 2021). Therefore, replacing the positively charged arginine residue with hydrophobic leucine could disrupt the structure of the amphipathic α-helix at the C-terminus of MAAP, thereby altering the functionality of this protein.

AAP is a scaffolding protein involved in AAV capsid assembly (Kuz et al., 2024). Moreover, the W28 position is located in the hydrophobic region of AAP and is found to be highly conservative among all AAV serotypes. Naumer et al. (2012) demonstrated that mutations within the region between W23 and W28 result in a substantial decline in assembly-promoting activity.

It is established that five amino acids, including N470, D271, N272, Y446, and W503, form a pocket at the base of the protrusions around the icosahedral 3-fold axes of symmetry (Bell et al., 2012). These amino acids are essential for galactose binding, which is the initial stage of AAV entry into the cell. As the mutations we introduced do not affect this region, but only the VP1/VP2 and VP1u regions, it can be concluded that the increase in transduction efficiency was achieved through improved intracellular trafficking and endosomal escape rather than through viral entry into the cell (Khanal et al., 2023).

Both SDS-PAGE and LC-MS/MS were used in this study to estimate the relative content of capsid proteins. Due to the varying sensitivity of proteins to staining and high error rates, SDS-PAGE does not allow for accurate analysis of the content of different proteins in a sample. However, semi-quantitative densitometry shows the general trend in the change in their ratio. Thus, an increase in the relative content of VP1 or VP2 was detected in the corresponding capsids (Supplementary Figure S3). To obtain a more detailed characterization, LC-MS/MS analysis was performed (Serrano et al., 2023). The LC-MS/MS is a reliable and precise method to identify different AAV serotypes. It enables the accurate measurement of the molecular weight of intact VP proteins, facilitating analysis of post-translational modifications or point substitutions, which could not be conducted with SDS-PAGE (Zhou and Wang, 2023; Jin et al., 2017; Lam et al., 2022; Zhang et al., 2021; Liu et al., 2020; Tiambeng et al., 2025). Furthermore, the migration of capsid proteins in the gel may be affected by polypeptide sequence and does not always correlate with their theoretical masses. For instance, AAV2 proteins, which have the highest calculated masses, demonstrate greater mobility in the gel than AAV5 and AAV8 proteins (Lam et al., 2022).

The analysis of intact viral particles by LC-MS/MS revealed characteristic changes in the stoichiometric ratio of VP1, VP2, and VP3 structural proteins in the samples studied (Table 3). In wild-type capsids, the VP1:VP2:VP3 ratio was 0.7:0.8:10. In the AAV9-VP1-2 samples, an increase in the VP1 to VP2 ratio was observed, whereas in the AAV9-VP1-2 variant, VP2 predominated.

**TABLE 3:** Stoichiometric ratios of capsid proteins determined by LC-MS/MS.

Sample	VP1	VP2	VP3
WT	0.7	0.8	10.0
AAV9-VP1-2	0.8	0.4	10.0
AAV9-VP2-2	0.4	0.7	10.0

Despite the difference in absolute values, analyses employing two methods–SDS-PAGE with densitometry and LC-MS/MS–revealed a fundamentally consistent trend: the AAV9-VP1-2 and AAV9-VP2-2 capsid variants demonstrated opposite changes in the VP1/VP2 ratio compared to wild-type capsids. The observed differences in absolute values can be attributed to the varying sensitivity of the methods to the unique features of electrophoretic mobility and staining efficiency of individual capsid proteins.

The main outcome of this study is the proof of concept that a simple method can be used to modify the physical and therapeutic properties of recombinant AAV during development. This modification strategy also opens up new opportunities for the study of AAV properties, which has already demonstrated its efficacy in gene therapy. The tested approach is highly similar to the conventional triple-plasmid transfection method. However, it enables the development of vectors with enhanced transduction efficiency and does not necessitate any additional stages during the development process. We believe that combining the results of our study with other modern approaches to AAV capsid research (Ling et al., 2024; Meng et al., 2024) could improve the safety and efficacy of gene therapy. Furthermore, the utilization of independent VP2 expression facilitates the linkage of its N-terminus within an open reading frame with other proteins and peptides without compromising the amino acid sequence of VP1.

Methodology described here could be applied to many gene therapy applications, including the development of effective capsids for treatment of retinopathies, neuropathies, and metabolic disorders (Tian et al., 2023). In the future studies, the potential for upscaling the technology to facilitate the industrial production of vectors in a bioreactor format will be evaluated. This technique of capsid modification could also be used to conjugate the N-terminus of VP2 with cell-penetrating peptides, thereby modifying the tropism of AAV9.

## Data Availability

The original contributions presented in the study are included in the article/Supplementary Material, further inquiries can be directed to the corresponding author.
