# Supplementary material for: Altering VP1 and VP2 expression in trans affects the transduction efficiency of AAV9
Source: Front Bioeng Biotechnol. 2026 Feb 26;14:1753246. doi: 10.3389/fbioe.2026.1753246 (PMC12979495; doi:10.3389/fbioe.2026.1753246)
Supplement: Supplementary file 1 [file DataSheet1.pdf]

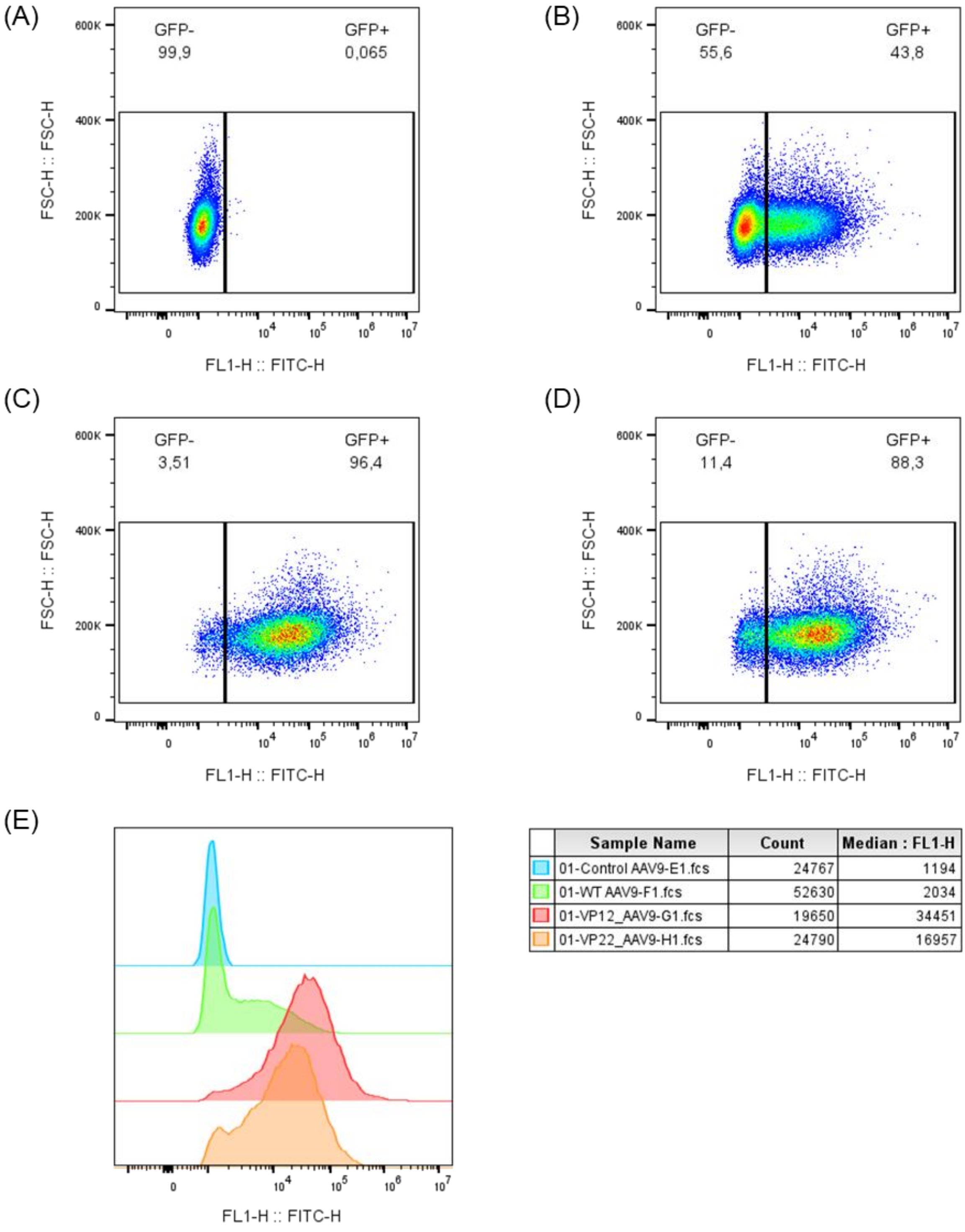


| | Sample Name | Count | Median : FL1-H |
|---|---|---|---|
| | 01-Control AAV9-E1.fcs | 24767 | 1194 |
| | 01-WT AAV9-F1.fcs | 52630 | 2034 |
| | 01-VP12_AAV9-G1.fcs | 19650 | 34451 |
| | 01-VP22_AAV9-H1.fcs | 24790 | 16957 |

Figure S1. Flow cytometry data 72 hours after transduction of HEK293TN cells at a dose of 100,000 viral genomes per cell. The relative content of GFP+ cells is shown among single live cells that were not transduced (A), transduced with wild-type capsids (B), AAV9-VP-1-2 capsids (C) and AAV9-VP-2-2 capsids (D). (E) Median FITC-H values for all capsid variants.

(A)

VP1

MAADGYLPDWLEDNLSEGIREWWALKPGAPQPKANQQHQDNARGLVLPGYKYLGPGNGLDKGEPVNAADAALEHDKAYDQQLKAGDNPYLKYNHADAEFQERLKEDTSFGGNLGRAVFQAKKRLL
1
EPLGLVEEAAKTAPGKKRPVEQSPQEPDSSAGIGKSGAQPAKKRLNFGQTGDTESVPDPQPIGEPPAAPSGVGSLTMASGGGAPVADNNEGADGVGSSSGNWHCDSQWLGDRVITTSTRTWALPTY
138 203
NNHLYKQISNSTSGGSSNDNAYFGYSTPWGYFDFNRFHCHFSPRDWQRLINNNWGFRPKRLNFKLFNIQVKEVTDNNGVKTIANNLTSTVQVFTDSDYQLPYVLGSAHEGCLPPFPADVFMIPQYGYLT
LNDGSQAVGRSSFYCLEYFPSQMLRTGNNFQFSYEFENVPFHSSYAHSQSLDRLMNPLIDQYLYYLSKTINGSGQNQQTLKFSVAGPSNMAVQGRNYIPGPSYRQQRVSTTVTQNNNSEFAWPGASS
WALNGRNSLMNPGPAMASHKEGEDRFFPLSGSLIFGKQGTGRDNVDADKVMITNEEEIKTTNPVATESYGQVATNHQSAQAQAQTGWVQNQGILPGMVWQDRDVYLQGPIWAKIPHTDGNFHPSPLM
GGFGMKHPPPQILIKNTPVPADPPTAFNKDKLNSFITQYSTGQVSVEIEWELQKENSKRWNPEIQYTSNYYKSNNVEFAVNTEGVYSEPRPIGTRYLTRNL

VP2

TAPGKKRPVEQSPQEPDSSAGIGKSGAQPAKKRLNFGQTGDTESVPDPQPIGEPPAAPSGVGSLTMASGGGAPVADNNEGADGVGSSSGNWHCDSQWLGDRVITTSTRTWALPTYNNHLYKQISNS
138 203
TSGGSSNDNAYFGYSTPWGYFDFNRFHCHFSPRDWQRLINNNWGFRPKRLNFKLFNIQVKEVTDNNGVKTIANNLTSTVQVFTDSDYQLPYVLGSAHEGCLPPFPADVFMIPQYGYLTLNDGSQAVGR
SSFYCLEYFPSQMLRTGNNFQFSYEFENVPFHSSYAHSQSLDRLMNPLIDQYLYYLSKTINGSGQNQQTLKFSVAGPSNMAVQGRNYIPGPSYRQQRVSTTVTQNNNSEFAWPGASSWALNGRNSLM
NPGPAMASHKEGEDRFFPLSGSLIFGKQGTGRDNVDADKVMITNEEEIKTTNPVATESYGQVATNHQSAQAQAQTGWVQNQGILPGMVWQDRDVYLQGPIWAKIPHTDGNFHPSPLMGGFGMKHPPP
QILIKNTPVPADPPTAFNKDKLNSFITQYSTGQVSVEIEWELQKENSKRWNPEIQYTSNYYKSNNVEFAVNTEGVYSEPRPIGTRYLTRNL

VP3

MASGGGAPVADNNEGADGVGSSSGNWHCDSQWLGDRVITTSTRTWALPTYNNHLYKQISNSTSGGSSNDNAYFGYSTPWGYFDFNRFHCHFSPRDWQRLINNNWGFRPKRLNFKLFNIQVKEVTDN
203
NGVKTIANNLTSTVQVFTDSDYQLPYVLGSAHEGCLPPFPADVFMIPQYGYLTLNDGSQAVGRSSFYCLEYFPSQMLRTGNNFQFSYEFENVPFHSSYAHSQSLDRLMNPLIDQYLYYLSKTINGSGQNQ
QTLKFSVAGPSNMAVQGRNYIPGPSYRQQRVSTTVTQNNNSEFAWPGASSWALNGRNSLMNPGPAMASHKEGEDRFFPLSGSLIFGKQGTGRDNVDADKVMITNEEEIKTTNPVATESYGQVATNHQ
SAQAQAQTGWVQNQGILPGMVWQDRDVYLQGPIWAKIPHTDGNFHPSPLMGGFGMKHPPPQILIKNTPVPADPPTAFNKDKLNSFITQYSTGQVSVEIEWELQKENSKRWNPEIQYTSNYYKSNNVEF
AVNTEGVYSEPRPIGTRYLTRNL

(B)

VP1

MAADGYLPDWLEDNLSEGIREWWALKPGAPQPKANQQHQDNARGLVLPGYKYLGPGNGLDKGEPVNAADAALEHDKAYDQQLKAGDNPYLKYNHADAEFQERLKEDTSFGGNLGRAVFQAKKRLL
1
EPLGLVEEAAKAAPGKKRPVEQSPQEPDSSAGIGKSGAQPAKKRLNFGQTGDTESVPDPQPIGEPPAAPSGVGSLTLASGGGAPVADNNEGADGVGSSSGNWHCDSQWLGDRVITTSTRTWALPTY
138 203
NNHLYKQISNSTSGGSSNDNAYFGYSTPWGYFDFNRFHCHFSPRDWQRLINNNWGFRPKRLNFKLFNIQVKEVTDNNGVKTIANNLTSTVQVFTDSDYQLPYVLGSAHEGCLPPFPADVFMIPQYGYLT
LNDGSQAVGRSSFYCLEYFPSQMLRTGNNFQFSYEFENVPFHSSYAHSQSLDRLMNPLIDQYLYYLSKTINGSGQNQQTLKFSVAGPSNMAVQGRNYIPGPSYRQQRVSTTVTQNNNSEFAWPGASS
WALNGRNSLMNPGPAMASHKEGEDRFFPLSGSLIFGKQGTGRDNVDADKVMITNEEEIKTTNPVATESYGQVATNHQSAQAQAQTGWVQNQGILPGMVWQDRDVYLQGPIWAKIPHTDGNFHPSPLM
GGFGMKHPPPQILIKNTPVPADPPTAFNKDKLNSFITQYSTGQVSVEIEWELQKENSKRWNPEIQYTSNYYKSNNVEFAVNTEGVYSEPRPIGTRYLTRNL

VP2

TAPGKKRPVEQSPQEPDSSAGIGKSGAQPAKKRLNFGQTGDTESVPDPQPIGEPPAAPSGVGSLTMASGGGAPVADNNEGADGVGSSSGNWHCDSQWLGDRVITTSTRTWALPTYNNHLYKQISNS
138 203
TSGGSSNDNAYFGYSTPWGYFDFNRFHCHFSPRDWQRLINNNWGFRPKRLNFKLFNIQVKEVTDNNGVKTIANNLTSTVQVFTDSDYQLPYVLGSAHEGCLPPFPADVFMIPQYGYLTLNDGSQAVGR
SSFYCLEYFPSQMLRTGNNFQFSYEFENVPFHSSYAHSQSLDRLMNPLIDQYLYYLSKTINGSGQNQQTLKFSVAGPSNMAVQGRNYIPGPSYRQQRVSTTVTQNNNSEFAWPGASSWALNGRNSLM
NPGPAMASHKEGEDRFFPLSGSLIFGKQGTGRDNVDADKVMITNEEEIKTTNPVATESYGQVATNHQSAQAQAQTGWVQNQGILPGMVWQDRDVYLQGPIWAKIPHTDGNFHPSPLMGGFGMKHPPP
QILIKNTPVPADPPTAFNKDKLNSFITQYSTGQVSVEIEWELQKENSKRWNPEIQYTSNYYKSNNVEFAVNTEGVYSEPRPIGTRYLTRNL

VP3

MASGGGAPVADNNEGADGVGSSSGNWHCDSQWLGDRVITTSTRTWALPTYNNHLYKQISNSTSGGSSNDNAYFGYSTPWGYFDFNRFHCHFSPRDWQRLINNNWGFRPKRLNFKLFNIQVKEVTDN
203
NGVKTIANNLTSTVQVFTDSDYQLPYVLGSAHEGCLPPFPADVFMIPQYGYLTLNDGSQAVGRSSFYCLEYFPSQMLRTGNNFQFSYEFENVPFHSSYAHSQSLDRLMNPLIDQYLYYLSKTINGSGQNQ
QTLKFSVAGPSNMAVQGRNYIPGPSYRQQRVSTTVTQNNNSEFAWPGASSWALNGRNSLMNPGPAMASHKEGEDRFFPLSGSLIFGKQGTGRDNVDADKVMITNEEEIKTTNPVATESYGQVATNHQ
SAQAQAQTGWVQNQGILPGMVWQDRDVYLQGPIWAKIPHTDGNFHPSPLMGGFGMKHPPPQILIKNTPVPADPPTAFNKDKLNSFITQYSTGQVSVEIEWELQKENSKRWNPEIQYTSNYYKSNNVEF
AVNTEGVYSEPRPIGTRYLTRNL

(C)

VP1

MAADGYLPDWLEDNLSEGIREWWALKPGAPQPKANQQHQDNARGLVLPGYKYLGPGNGLDKGEPVNAADAALEHDKAYDQQLKAGDNPYLKYNHADAEFQERLKEDTSFGGNLGRAVFQAKKRLL
1
EPLGLVEEAAKAAPGKKRPVEQSPQEPDSSAGIGKSGAQPAKKRLNFGQTGDTESVPDPQPIGEPPAAPSGVGSLTMASGGGAPVADNNEGADGVGSSSGNWHCDSQWLGDRVITTSTRTWALPTY
138 203
NNHLYKQISNSTSGGSSNDNAYFGYSTPWGYFDFNRFHCHFSPRDWQRLINNNWGFRPKRLNFKLFNIQVKEVTDNNGVKTIANNLTSTVQVFTDSDYQLPYVLGSAHEGCLPPFPADVFMIPQYGYLT
LNDGSQAVGRSSFYCLEYFPSQMLRTGNNFQFSYEFENVPFHSSYAHSQSLDRLMNPLIDQYLYYLSKTINGSGQNQQTLKFSVAGPSNMAVQGRNYIPGPSYRQQRVSTTVTQNNNSEFAWPGASS
WALNGRNSLMNPGPAMASHKEGEDRFFPLSGSLIFGKQGTGRDNVDADKVMITNEEEIKTTNPVATESYGQVATNHQSAQAQAQTGWVQNQGILPGMVWQDRDVYLQGPIWAKIPHTDGNFHPSPLM
GGFGMKHPPPQILIKNTPVPADPPTAFNKDKLNSFITQYSTGQVSVEIEWELQKENSKRWNPEIQYTSNYYKSNNVEFAVNTEGVYSEPRPIGTRYLTRNL

VP2

TAPGKKRPVEQSPQEPDSSAGIGKSGAQPAKKRLNFGQTGDTESVPDPQPIGEPPAAPSGVGSLTLASGGGAPVADNNEGADGVGSSSGNWHCDSQWLGDRVITTSTRTWALPTYNNHLYKQISNST
138 203
SGGSSNDNAYFGYSTPWGYFDFNRFHCHFSPRDWQRLINNNWGFRPKRLNFKLFNIQVKEVTDNNGVKTIANNLTSTVQVFTDSDYQLPYVLGSAHEGCLPPFPADVFMIPQYGYLTLNDGSQAVGRS
SFYCLEYFPSQMLRTGNNFQFSYEFENVPFHSSYAHSQSLDRLMNPLIDQYLYYLSKTINGSGQNQQTLKFSVAGPSNMAVQGRNYIPGPSYRQQRVSTTVTQNNNSEFAWPGASSWALNGRNSLMN
PGPAMASHKEGEDRFFPLSGSLIFGKQGTGRDNVDADKVMITNEEEIKTTNPVATESYGQVATNHQSAQAQAQTGWVQNQGILPGMVWQDRDVYLQGPIWAKIPHTDGNFHPSPLMGGFGMKHPPPQ
ILIKNTPVPADPPTAFNKDKLNSFITQYSTGQVSVEIEWELQKENSKRWNPEIQYTSNYYKSNNVEFAVNTEGVYSEPRPIGTRYLTRNL

VP3

MASGGGAPVADNNEGADGVGSSSGNWHCDSQWLGDRVITTSTRTWALPTYNNHLYKQISNSTSGGSSNDNAYFGYSTPWGYFDFNRFHCHFSPRDWQRLINNNWGFRPKRLNFKLFNIQVKEVTDN
203
NGVKTIANNLTSTVQVFTDSDYQLPYVLGSAHEGCLPPFPADVFMIPQYGYLTLNDGSQAVGRSSFYCLEYFPSQMLRTGNNFQFSYEFENVPFHSSYAHSQSLDRLMNPLIDQYLYYLSKTINGSGQNQ
QTLKFSVAGPSNMAVQGRNYIPGPSYRQQRVSTTVTQNNNSEFAWPGASSWALNGRNSLMNPGPAMASHKEGEDRFFPLSGSLIFGKQGTGRDNVDADKVMITNEEEIKTTNPVATESYGQVATNHQ
SAQAQAQTGWVQNQGILPGMVWQDRDVYLQGPIWAKIPHTDGNFHPSPLMGGFGMKHPPPQILIKNTPVPADPPTAFNKDKLNSFITQYSTGQVSVEIEWELQKENSKRWNPEIQYTSNYYKSNNVEF
AVNTEGVYSEPRPIGTRYLTRNL

Figure S2. Amino acid sequences of VP1, VP2, and VP3 proteins of AAV9-WT (A), AAV9-VP-1-2 (B), and AAV9-VP-2-2 (C) capsids.

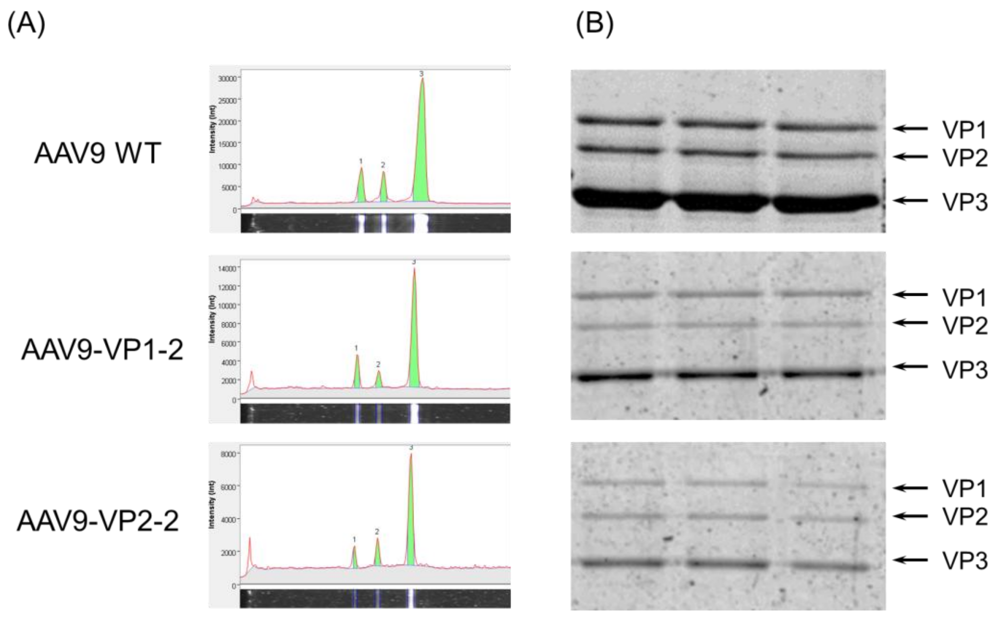


Figure S3. Increased VP1 and VP2 content, as determined by quantitative densitometry. (A) Graphs showing the intensity of the capsid structural protein bands on the electrophoregram. (B) Capsid protein electrophoresis performed in three replicates. Legend: AAV9 WT — wild-type capsids; AAV9-VP1-2 — capsids with increased VP1 content; AAV9-VP2-2 — capsids with increased VP2 content.
